# Vitamins D and K jointly protect against osteoarthritis via regulating OSCAR during osteoclastogenesis

**DOI:** 10.1016/j.jot.2025.03.018

**Published:** 2025-05-12

**Authors:** Yang Zhao, Qianhua Ou, Hong Huang, Delong Li, Jianmao Chen, Song Xue, Zuoqing Zhou, Guangfeng Ruan, Changhai Ding

**Affiliations:** a Clinical Research Centre, Zhujiang Hospital, Southern Medical University, Guangzhou, 510000, Guangdong, China; b Department of Intensive Care Unit, Zhongshan City People's Hospital, Zhongshan, 528403, Guangdong, China; c Department of Orthopedics, The Third Affiliated Hospital, Southern Medical University, Guangzhou, 510000, Guangdong, China; d Department of Orthopedics, Foshan First People's Hospital, Foshan, 528000, Guangdong, China; e Department of Sports Medicine and Rehabilitation, Peking University Shenzhen Hospital, Shenzhen Peking University-The Hong Kong University of Science and Technology Medical Center, Shenzhen, 518036, China; f Department of Orthopedics, The First Affiliated Hospital, Shaoyang University, Shaoyang, 410000, Hunan, China; g Clinical Research Centre, Guangzhou First People's Hospital, South China University of Technology, Guangzhou, 510000, Guangdong, China; h Menzies Institute for Medical Research, University of Tasmania, Hobart, Tasmania, 7000, Australia

**Keywords:** Vitamin D, Vitamin K, Osteoarthritis, Subchondral bone remodeling, Pain, Osteoclast, OSCAR

## Abstract

**Objective:**

The effects of vitamins D and K on osteoarthritis (OA) progression remain ambiguous, particularly in its subtype, osteoporotic OA (OPOA), where aberrant activation of osteoclasts exacerbates subchondral bone remodeling. This study aimed to investigate the effect of 1,25-dihydroxyvitamin D3 (calcitriol) and menaquinone-4 (MK4) on OA and OPOA progression and explore their combined mechanisms in osteoclastogenesis inhibition.

**Methods:**

Therapeutic effects of calcitriol and MK4 were evaluated in OA and OPOA models induced by medial meniscus destabilization (DMM) and bilateral ovariectomy (OVX). In vitro analyses assessed their impact on chondrocyte degradation and osteoclastogenesis. RNA sequencing of preosteoclasts elucidated the vitamins' anti-osteoclastogenic mechanisms.

**Results:**

Combined administration of calcitriol and MK4 significantly attenuated cartilage degradation in OA and OPOA mouse models, though direct effects on chondrocyte degradation were limited. Importantly, calcitriol and MK4 jointly suppressed osteoclastogenesis in vivo and in vitro, ameliorating subchondral remodeling and reducing pain levels in OPOA mice. Mechanistically, osteoclast-associated receptor (OSCAR) mediated their anti-osteoclastogenic effects.

**Conclusions:**

Calcitriol and MK4 confer enhanced benefits on OA and OPOA progression through OSCAR-mediated osteoclastogenesis inhibition in preosteoclasts.

**The Translational potential of this article:**

This study demonstrates vitamins D and K as dual-action agents inhibiting osteoclastogenesis and normalizing subchondral bone remodeling both in OA and OPOA models, making it a potential therapeutic alternative for the disease.

## Graphical abstract

**Figure:**
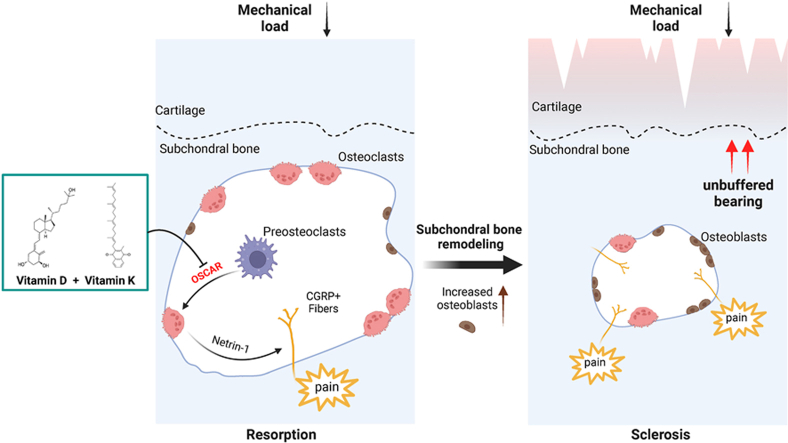

## 1 Introduction

Osteoarthritis (OA) is a prevalent disabling disease among the elderly, which results in joint pain, stiffness, and disability. Approximately 250 million people worldwide suffer from symptomatic OA [1]. As a whole joint disease, the pathological features of OA mainly involve cartilage degradation, synovitis, and subchondral bone remodeling [2,3]. Multiple strategies targeting cartilage repair, extracellular matrix protease, inflammation, and other bioactive reagents have been investigated for OA treatment, although their efficacy and safety are still unclear [4]. Currently, no treatment options are available to halt the progression of OA, except for arthroplasty in the advanced stage [5].

Subchondral bone remodeling is essential to OA progression [6]. In healthy joints, subchondral bone nourishes cartilage and cushions against joint impact [7]. However, during the early stage of OA, aberrantly enhanced osteoclastogenesis in the subchondral bone is observed, leading to a deteriorated bone turnover and released transforming growth factor-β1 (TGF-β1) from the bone matrix [8]. In the late stage of OA, subsequently, mesenchymal stem cells are excessively recruited and differentiate into osteoblasts by TGF-β1 signaling, which results in accelerated bone formation, thickened subchondral bone microstructure, and impaired buffering function, thus aggravating cartilage degradation [9]. Moreover, overactivated osteoclasts secrete Netrin-1, which stimulates the growth of sensory nerves into the subchondral bone marrows, leading to increased pain sensitivity of the joints [10]. As documented osteoclast inhibitors, bisphosphonates reduce subchondral bone turnover, alleviate cartilage deterioration, and target bone marrow lesions and vascularization in animal studies [11]. Additionally, a review highlighted the osteoclast-mediated benefits of bone-acting agents, including bisphosphonates, parathyroid hormone analogs, and denosumab, in post-menopausal OA [12]. Therefore, it was proposed that regulation of aberrant subchondral bone remodeling by suppressing overactivated osteoclastogenesis could be a promising approach for OA treatment [13].

As a common and debilitating condition like OA, osteoporosis (OP) also frequently affects the elderly, particularly postmenopausal females [14]. The hallmark feature of OP, increased bone resorption, was also an important pathological change in the subchondral bone during the early stage of OA [15], which indicates a possible connection between OP and OA in pathogenesis. Osteoporotic OA (OPOA) has been recognized as a subtype of OA characterized by excessive osteoclast activity and aggravated subchondral bone remodeling with accelerated cartilage degradation [16,17].

Vitamins D and K are both fat-soluble nutrients playing essential roles in multiple physical activities, including skeletal metabolism, immune regulation, and blood coagulation [18,19]. Currently, vitamins D and K have been extensively utilized in the treatment of OP. However, the clinical significance of vitamins D and K in relation to OA and its subtype, OPOA, remains unclear. Current evidence from limited clinical research presents several points of controversy. On one hand, insufficient serum levels of vitamins D and K, and the usage of vitamin K antagonist anticoagulant are associated with an increased incidence and progression of OA, while adequate levels or sufficient intake of these vitamins are correlated with improved knee structure and symptoms [[20], [21], [22], [23]]. On the other hand, randomized clinical trials (RCTs) conducted globally have shown that vitamin D is ineffective in preventing cartilage loss, although it does reduce the Western Ontario and McMaster Universities Arthritis Index (WOMAC) pain score and enhance function in patients with knee OA [24]. With respect to vitamin K, only one RCT has been conducted concerning OA. Neogi et al. reported that phylloquinone (vitamin K1) supplementation did not lead to improvements in radiographic findings of hand OA, even among patients with insufficient baseline levels of vitamin K [25]. Overall, the limited efficacy of single administration of vitamins D or K has impeded their advancement as potential disease-modifying osteoarthritis drugs (DMOADs).

Recently, researchers have begun to examine the two vitamins in conjunction. Shea et al. reported that individuals with adequate levels of vitamins D and K demonstrated superior lower limb function compared to those deficient in vitamin D, vitamin K, or both, based on data analysis from two large OA cohorts [26]. Furthermore, it has been reported that specific vitamin K-dependent proteins, such as osteocalcin (OCN) and matrix Gla protein (MGP), can be upregulated by vitamin D and carboxylated by vitamin K, thereby promoting mineral deposition in osteoblasts and inhibiting arterial calcification [27,28]. These findings indicate a potential functional interaction between the two vitamins. However, the extent to which combined supplementation of vitamins D and K exerts a therapeutic effect on OA, and its subtype OPOA, or whether these two vitamins are mutually essential for each other's functional outcomes, remains largely unknown.

In this study, we aimed to investigate the specific roles of 1,25-dihydroxyvitamin D3 (calcitriol, here after referred to as vitamin D) and menaquinone-4 (MK4, here after referred to as vitamin K), two commonly utilized forms of vitamins D and K, respectively, in the progression of OA and OPOA, as well as their underlying mechanisms. We demonstrated that combined treatment of vitamin D and K effectively ameliorated cartilage degradation and pain level in vivo, not through direct preservation of chondrocyte degradation, but through jointly inhibiting osteoclastogenesis, thereby normalizing subchondral bone remodeling and sensory innervation. Mechanistically, we identified osteoclast-associated receptor (OSCAR) as an essential mediator for the joint effect of vitamins D and K on osteoclastogenic inhibition. Our study could provide novel insights into the clinical significance of vitamins D and K for OA and OPOA patients.

## 2 Materials and methods

### 2.1 Reagents and materials

Calcitriol (HY-10002) and Menaquinone-4 (HY-B2156) were purchased from MedChemExpress (New Jersey, USA). Dulbecco's modified eagle's medium: F12 (DMEM/F12), alpha-modified eagle's medium (α-MEM), and fetal bovine serum (FBS) were obtained from Invitrogen (California, USA). Penicillin-Streptomycin Solution and 0.25 % trypsin were purchased from Beyotime (Shanghai, China). Recombinant human interleukin 1β (IL-1β) (CG93) and recombinant mouse macrophage-colony stimulating factor (M-CSF) (CB34) were purchased from Novoprotein (Jiangsu, China). Recombinant mouse receptor activator of nuclear factor-κB ligand (RANKL) (462-TEC) was purchased from R&D Systems (Minnesota, USA). Polyvinylidene fluoride (PVDF) membranes were purchased from Millipore (Massachusetts, USA). Safranin O/fast green (G1371), hematoxylin (G1120), 4 % paraformaldehyde (PFA) (P1110), ethylene diamine tetra-acetic acid (EDTA) decalcifying solution (E1171), tartrate-resistant acid phosphatase (TRAP) stain kit (G1492), and Goldner's trichrome stain kit (G3550) were purchased from Solarbio (Beijing, China). Cell Counting Kit-8 (CCK-8) (C0038) was supplied by Beyotime (Shanghai, China).

### 2.2 OA and OPOA mouse models

Male and female C57BL/6J mice were obtained from Baishitong Corporation (Beijing, China). 12-week-old mice were used for the drug treatment, followed by OA and OPOA modeling. Mouse experiments were conducted in a specific pathogen-free laboratory. All mice received vitamin D and K deficient chow (Trophic Animal Feed High-Tech Co., Ltd, China) throughout the experiment.

For OA models and drug intervention, male mice were randomly assigned to five groups (n = 12 per group) based on a random number table: (1) SHAM group, receiving sham surgery; (2) DMM group, receiving destabilization of medial meniscus surgery (DMM); (3) DMM + Vit D group, receiving DMM and calcitriol (20 μg/kg every other day); (4) DMM + Vit K group, receiving DMM and MK4 (50 μg/kg every other day); and (5) DMM + Vit D + K group, receiving both calcitriol and MK4 as scheduled. Mice in different groups were pre-treated with indicated doses of calcitriol and MK4 by gavage for two weeks before receiving DMM as previously described [29]. After anesthesia with isoflurane inhalation, a longitudinal incision was made on the right knee to transect the medial meniscus tibial ligament under a surgical microscope. One day post-DMM, treatment continued until harvest. After five or ten weeks of treatment, mice were euthanized and knees were sampled for further analysis.

The OVX + DMM model combines systemic estrogen deficiency (OVX) with localized joint instability (DMM) to simulate OPOA pathophysiology. This dual-intervention approach induces accelerated joint degeneration, subchondral bone remodeling, and systemic bone loss, thereby enhancing both pathological relevance and translational potential. For OPOA models and drug intervention, female mice were randomly assigned to six groups (n = 18 per group) based on a random number table: (1) SHAM group, receiving sham surgery; (2) DMM group, receiving DMM; (3) OVX + DMM group, receiving both DMM and bilateral ovariectomy (OVX); (4) OVX + DMM + Vit D group, receiving OVX + DMM and calcitriol (20 μg/kg every other day); (5) OVX + DMM + Vit K group, receiving OVX + DMM and MK4 (50 μg/kg every other day); and (6) OVX + DMM + Vit D + K group, receiving both calcitriol and MK4 as scheduled. Two weeks prior to DMM, mice in different groups were pre-treated with indicated doses of calcitriol and MK4 and underwent OVX to induce osteoclast activation as previously described [30]. After anesthesia with isoflurane inhalation, a longitudinal incision was made on the back for ligation of fallopian tubes followed by removal of ovaries. One day post-OVX, treatment continued until harvest. Knee joints were collected at two, six, and ten weeks post-DMM for further analysis.

Finally, the sample size was calculated based on a previously described principle [31]. Briefly, the sample size was decided by the “resource equation”: E = (the total number of experimental units) – (the number of treatment groups). The value of E was chosen to be between 10 and 20. In our study, the experiment was divided into five (OA model) or six (OPOA model) treatment groups, and the mice were euthanized in two time points. Thus, there were 10 and 12 treatment factors for OA and OPOA model, respectively. Therefore, four to six mice per group were required at least.

### 2.3 Human cartilage

This study was approved by the Ethics Committee of Zhujiang Hospital, Southern Medical University (2019-KY-022-03). The articular cartilage samples were obtained from patients undergoing total knee replacement for end-stage OA (excluding those with inflammatory or infectious arthritis) at the Department of Orthopedics, Zhujiang Hospital, Southern Medical University. We followed all ethical guidelines and informed all patients of the procedures of this study. All patients signed informed consent prior to surgery and their basic clinical data were collected (Table S3).

### 2.4 Cell isolation and culture

Cartilage samples for primary chondrocyte isolation were harvested from visually intact areas of the tibial plateau and femoral condyle, avoiding severely damaged or fibrillated regions to ensure the isolation of viable chondrocytes. Primary human chondrocytes were digested and isolated according to the protocol previously reported [32]. Cartilage tissue was washed three times with sterile PBS containing 1 % Penicillin-Streptomycin and then carved into fragments (0.5 mm^3^). The chopped pieces were soaked in 0.25 % trypsin (containing 0.02 % EDTA) in a 37 °C shaker (100 rpm) for 30 min, followed by incubation with 0.2 % collagenase type II in a 37 °C thermostatic oscillator for 24 h. Primary chondrocytes were obtained by gradient centrifugation of digestive fluid. Chondrocytes were cultured in a 10 cm culture dish using DMEM/F12 medium (containing 10 % FBS and 1 % Penicillin-Streptomycin) for three days and were passaged at 80 % confluence.

Primary mouse bone marrow-derived macrophages (BMDMs) were isolated as previously described [33]. Briefly, 10-week-old C57BL/6J mice were euthanized, and bilateral legs were collected by sterile operation. After opening both terminals of the tibias and femurs, bone marrow was flushed into a culture dish using α-MEM (containing 10 % FBS and 1 % Penicillin-Streptomycin) and was incubated for 12 h. Subsequently, the non-adherent cells were collected and cultured with α-MEM containing 10 % FBS, 1 % Penicillin-Streptomycin, and M-CSF (20 ng/ml) to promote macrophage adhesion and proliferation.

### 2.5 Cell viability assay

Primary human chondrocytes (1 × 10^4^/well) and BMDMs (1 × 10^4^/well) were cultured separately in 96-well plates and then incubated with vitamin D or K at incremental concentrations for 48 h. Cells were washed with phosphate-buffered saline (PBS) twice and then incubated with culturing medium containing CCK-8 solution (10 μL) for 1 h (37 °C, 5 % CO_2_). The absorbance of the medium at 450 nm was then measured by a microplate reader and analyzed.

### 2.6 Osteoclastogenesis assay

For osteoclastogenesis, cells were incubated with M-CSF (20 ng/mL) and RANKL (100 ng/mL) in the presence or absence of the indicated doses of vitamins D and K or indicated treatments for four to six days until formed osteoclasts appeared. Next, cells were washed with PBS and fixed in 4 % PFA for 30 min, then TRAP staining was conducted with a kit, followed by image acquisition using a microscope (Leica, Germany), and TRAP-positive multinucleated cells were counted with the assistance of Image-pro plus software (USA) and differences between groups was further analyzed.

### 2.7 Osteoclast resorption activity (pit assay)

For osteoclast resorption activity assay, BMDMs were seeded on hydroxyapatite-coated plates (CLS3989; Corning). The cells were treated with M-CSF (20 ng/ml) and RANKL (100 ng/ml) in the presence or absence of the indicated doses of vitamins D and K or indicated treatments for three to five days. Cells were detached from the plate by washing buffer and were gently washed away, and images of hydroxyapatite resorption pits were captured by a light microscope (Leica, Germany) and the areas of the pits were measured by ImageJ software (USA).

### 2.8 Analysis of real-time quantitative polymerase chain reaction (RT-qPCR)

Total RNA was extracted from primary chondrocytes and BMDMs with TRIZOL (AGbio, China). 300–500 ng RNA was then reverse-transcribed into complementary DNA (cDNA) using reverse transcription reagents (AGbio, China). mRNA expression levels were detected using SYBR PremixExTaq (AGbio, China) by CFX connect (Bio-rad, USA). Glyceraldehyde-3-phosphate dehydrogenase (GAPDH) was set as the reference to analyze relative gene expression of each target based on 2-ΔΔCT method. Primer sequences of each gene are listed in Table S1.

### 2.9 Western blotting (WB) analysis and antibodies

Cultured cells were washed with pre-cooled PBS and lysed with Radio Immunoprecipitation Assay (RIPA) lysis buffer (containing 1 % phenylmethylsulfonyl fluoride). The collected proteins were quantified and denatured by being boiled in a metal bath with sodium dodecyl sulfate-polyacrylamide gel electrophoresis (SDS-PAGE) loading buffer (Beyotime, China). 20 μg of the protein per sample was subjected to electrophoresis and transferred onto PVDF membranes. Antibodies against glyceraldehyde-phosphate dehydrogenase (GAPDH) (1:5000; 60004-1-Ig, Proteintech), β-Tubulin (1:5000; 10094-1-AP, Proteintech), matrix metalloproteinase 13 (MMP13) (1:1000; ab39012, abcam), A disintegrin and metalloproteinase with thrombospondin motifs 5 (ADAMTS5) (1:1000; ab41037, abcam), type II Collagen alpha 1 (COL2A1) (1:1000; ab34712, abcam), aggrecan (ACAN) (1:1000; 13880-1-AP, Proteintech), Cathepsin K (CTSK) (1:1000; ab19027, abcam), C-FOS (1:1000; 2250, Cell Signaling Technology), Nuclear factor of activated T-cells, cytoplasmic 1 (NFATC1) (1:1000; 8032, Cell Signaling Technology), Netrin-1 (1:1000; ER1913-65, HUABIO) and OSCAR (1:1000; MAB1633, R&D system) were incubated with the PVDF membranes in 4 °C, overnight. After incubation with primary antibodies, samples were incubated with corresponding rabbit or mouse horseradish peroxidase (HRP)-labeled secondary antibodies (1:5000; Proteintech). Ultimately, Immunoreactive bands were visualized with an ECL system (NCM biotech, China), captured with a chemiluminescence apparatus (Bio-rad, USA) and analyzed using ImageJ software (USA).

### 2.10 RNA sequencing analysis

BMDMs stimulated with M-CSF (20 ng/ml), RANKL (100 ng/ml), and calcitriol (1000 nM) and MK4 (40 μM) for 48 h were collected for total RNA extraction using TRIzol Reagent (AGBio, China) according the manufacturer's instructions. Then RNA samples were determined and quantified. A cDNA library constructed by the pooled RNA was sequenced run with Illumina NovaseqTM 6000 sequence platform. Using the Illumina paired-end RNA-seq approach, we sequenced the transcriptome, generating a total of millon 2 x 150 bp paired-end reads. Differential expression analysis was performed by edgeR software between groups. Kyoto Encyclopedia of Genes and Genomes (KEGG) pathway enrichment analysis of the screened differentially expressed genes was conducted under the assistance of a bio-information processing website: https://www.bioinformatics.com.cn.

### 2.11 RNA interference

Small interfering RNAs (siRNAs) for mouse OSCAR were designed and synthesized by Igebio Bio (Guangdong, China). siRNA sequences specific to the OSCAR gene are listed in Table S2. BMDMs were transfected with siRNA using Lipofectamine 3000 (Thermo Fisher Scientific, USA) following the manufacturer's instructions. Briefly, BMDMs were starved in Opti-MEM for 30 min, then were transfected with OSCAR siRNAs or control siRNA with Lipofectamine 3000. After 12 h, the medium was replaced with α-MEM (containing 10 % FBS and 20 ng/ml M-CSF). After 24 h, cells were treated with M-CSF (20 ng/ml) and RANKL (100 ng/ml) to induce osteoclastogenesis for another 48 h. Protein and RNA samples of each group were collected for further analysis.

### 2.12 OSCAR overexpression by lentivirus transduction

Lentivirus (pcSLenti-EF1-EGFP-P2A-Puro-CMV-OSCAR-3xFLAG-NPRE) was obtained from OBiO Technology (Shanghai, China) to achieve the overexpression of OSCAR in preosteoclasts. BMDMs were transduced with lentivirus in the presence of 10 μg/mL polybrene for 24 h on Day 0 (the first day when BMDMs were induced by M-CSF and RANKL). The cell density of BMDMs in a 6-well plate was 5 × 10^5^ cells per well. The final Multiplicity of Infections (MOI) was controlled to 8 according to preliminary experiments. After incubation with lentivirus and polybrene for 24 h, cells were cultured in α-MEM (containing 10 % FBS, 20 ng/ml M-CSF, and 100 ng/ml RANKL) in the presence or absence of indicated doses of vitamins D and K. After 48 h, samples were collected for further analyses.

### 2.13 Histological analysis

Mouse knee joint tissues were harvested and fixed in 4 % paraformaldehyde for 48 h, followed by decalcification in 10 % ethylene diamine tetra acetic acid (EDTA) for two weeks. Then, samples were embedded in paraffin and serially sectioned (4 μm thickness) in the sagittal view of the medial compartment of the knee joint. Each knee was embedded in the same orientation. Next, Safranin O and fast green, TRAP, and Goldner's trichrome staining were performed following the manufacturer's instructions. The images were captured using a light microscope (DM2500, Leica, Germany). The summed Osteoarthritis Research Society International (OARSI) scores of medial femoral condyle and medial tibial plateau cartilage, with a maximum score of 12, were assessed to quantify the severity of cartilage degradation. TRAP staining was used to indicate mature osteoclasts in the subchondral bone of mice. Goldner's trichrome staining was used to indicate the process of bone formation. The above-mentioned analyses were conducted by two independent researchers blinded to the grouping details.

### 2.14 Immunohistochemistry and immunofluorescence analysis

Immunohistochemistry was conducted following a standard protocol published previously [34]. Briefly, the slices were baked at 68 °C and then soaked in xylene and gradient alcohol. Antigen retrieval was conducted by bathing the sections in diluted sodium citrate/EDTA repairing solution at 62 °C overnight. Endogenous peroxidase was blocked with 3 % H_2_O_2_, and slices were blocked in immunostaining blocking buffer for 1 h. Then, sections were incubated using primary antibodies against MMP13 (1:200; ab39012, abcam), COL2A1 (1:200; ab34712, abcam), COLX (1:200; ab58632, abcam), and OSX (1:200; ab22552, abcam) overnight at 4 °C. The next day, the slices were washed with PBS, incubated with the corresponding secondary antibodies conjugated with horse radish peroxidase (1:200; ABclonal) for 1 h, and incubated with diaminobenzidine reagent. Images were then captured with a light microscope (Leica, Germany).

The expression of calcitonin gene-related peptide (CGRP) was detected by immunofluorescence following a standard protocol [35]. After antigen retrieval and blocking, sections were incubated with primary antibodies against CGRP (1:200; Abcam, ab81882) overnight at 4 °C and fluorescent Alexa Fluor-555-conjugated secondary antibody (1:500) for 1 h. Nuclei were stained with 4’,6-diamidino-2-phenylindole (DAPI). Finally, CGRP expression in the subchondral bones was visualized using a fluorescence microscope (Nikon, Ti2-E) and was quantified by ImageJ software. The above-mentioned analyses were conducted by two independent researchers blinded to the grouping details.

### 2.15 Von Frey tests

Von Frey filaments were employed to measure the paw withdrawal threshold (PWT) of the hind limbs of model mice. Briefly, mice were placed in cages with metal mesh bottoms for 30 min to adapt them to the testing environment. According to the baseline confirmed before the test, Von Frey filaments applied in this test were the following serial forces (gram = 0.6, 1.0, 1.4, 2.0, 4.0, 6.0), and 6.0 g was set as the cut-off threshold. Mechanical allodynia was tested according to the up-down method of Dixon [36] and was conducted at the same time weekly. The 0.6 g filament was acupunctured beneath the middle part of the right hind paw until it bent as a “S” shape for 3–5 s. If there was a negative response, the next higher force was applied. The next lower force was adopted if there was a positive response (mice suddenly withdrawing or licking their hind paw). Three extra tests were performed to exclude false reactions and move on to the next mouse. The interval between adjacent stimuli was at least 5 min.

### 2.16 Micro-CT analysis

All knee joint specimens were fixed in 4 % paraformaldehyde and scanned using a high-resolution micro-CT Scanner (ZKKS-MCT-Sharp scanner, CASkaisheng, China). The scanner was set to a voltage of 70 kV, current of 100 μA, exposure time of 100 ms, and resolution of 10 μm. Three-dimensional reconstruction and image visualization of the knee joint were operated with Micro-CT Analysis Software (Micro-CT Reconstruction, ZKKS-Micro-CT 4.1). The region of interest in the sample images covered the whole subchondral bone. The following structural parameters of subchondral bone were measured in scanned images: BMD, bone volume/tissue volume (BV/TV), and trabecular separation (Tb. Sp).

### 2.17 Statistical analysis

Data in this study are presented as the Mean ± standard deviation (SD) for at least triplicate samples. All detailed data are indicated in the relevant figure legends. The analyses were performed by the SPSS version 20.0 software (IBM Corporation. USA). Statistically significant differences in each group were evaluated by using the two-tailed Student's t-test, one-way analysis of variance (ANOVA) followed by post hoc multiple comparison (LSD test) or Mann–Whitney U test for comparisons between groups. *P* values less than 0.05 (two-sided) were considered statistically significant. Statistical graphs were drawn by GraphPad Prism 9.0 (GraphPad Software, USA).

## 3 Results

### 3.1 Vitamins D and K attenuated cartilage degradation after DMM

**Fig. 1:**
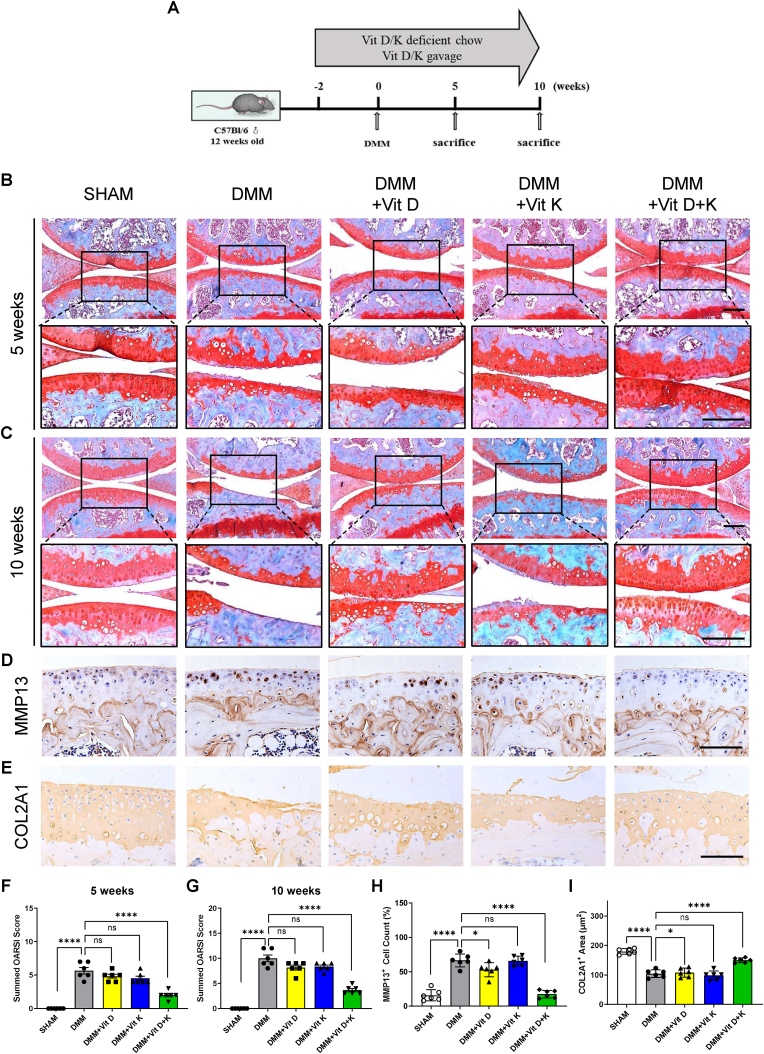
Vitamins D and K alleviate cartilage degradation in OA mouse models. A. Experimental scheme of vitamins D and K administration in OA mice. B-C. Safranin O/Fast green staining (SOFG) of articular cartilage 5 and 10 weeks after DMM. Scale bars, 100 μm. D-E. Immunohistochemistry analysis of MMP13 and COL2A1 in articular cartilage 10 weeks after DMM. Scale bars, 100 μm. F-G. Summed OARSI score based on SOFG at indicated times points (n = 6 mice per group). H. Ratio of MMP13 positive cells in the cartilage 10 weeks after DMM (n = 6 mice per group). I. COL2A1 positive area of cartilage 10 weeks after DMM (n = 6 mice per group). ns. None significant. ∗, *P* value < 0.05. ∗∗∗∗, *P* value < 0.0001.

To establish adequate therapeutic concentrations in murine models, thereby optimizing the pharmacological potential of vitamins D and K, the compounds were administered two weeks prior to DMM, and the mice were sacrificed after five or ten weeks of following modeling. This strategy aimed to evaluate the therapeutic effects of vitamins D and K during middle and late OA stages and their potential to halt disease progression (Fig. 1. A). Cartilage damage of OA mice after DMM at ten weeks was more severe compared to five weeks, and the combined vitamin D and K treatment reduced cartilage degradation at both time points, whereas vitamin D or vitamin K alone did not have any significant effect (Fig. 1 B-C). The summed OARSI scoring also indicated the therapeutic effect of vitamins D and K (Fig. 1 F, G). We further evaluated cartilage degradation in the OA mice by immunohistochemistry. The results showed that vitamins D and K treatment inhibited MMP13 and maintained COL2A1 expression in the cartilage ten weeks after DMM (Fig. 1 D-E, H-I). These findings demonstrated that vitamins D and K could jointly reduce cartilage damage in the DMM mouse models and that an interdependent relationship between vitamins D and K might exist.

### 3.2 Vitamins D and K exerted a limited effect on OA chondrocytes

We then investigated the role of vitamin D (Calcitriol) and vitamin K (Menaquinone-4) on OA phenotypes in chondrocytes. The viability of chondrocytes was significantly impaired at doses of 2000 nM for vitamin D, and 80 μM for vitamin K. Cell viability remained unaffected at doses of 1000 nM (vitamin D) and 40 μM (vitamin K) (Figure S1 A). Thus, the maximum doses of vitamins D and K for subsequent experiments were set at 1000 nM and 40 μM, respectively.

**Fig. 2:**
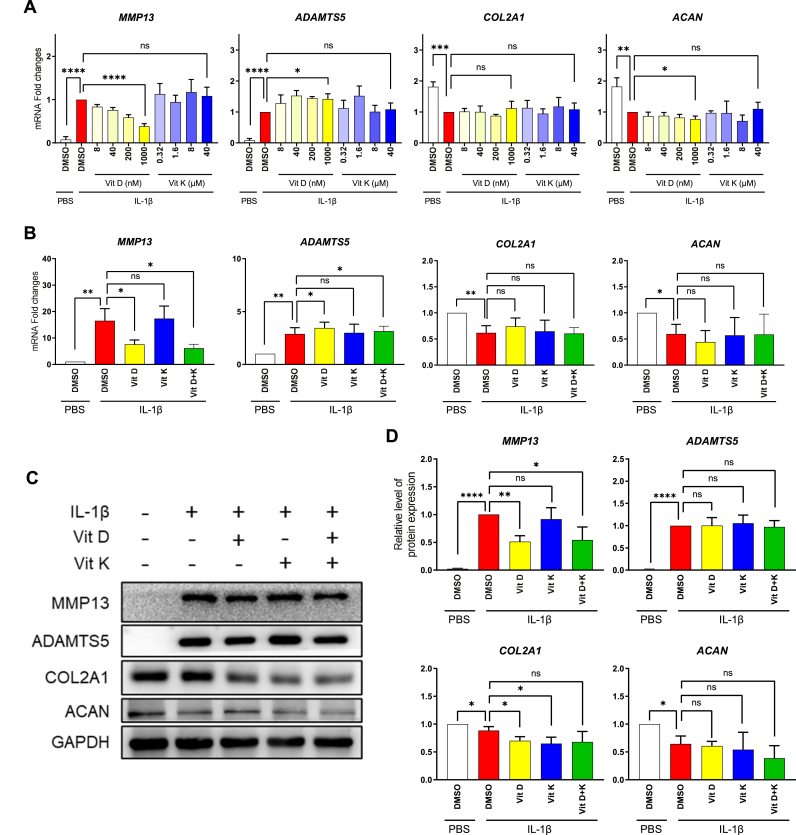
The role of vitamins D and K on chondrocyte degeneration. A. RT-qPCR analysis of MMP13, ADAMTS5, COL2A1, and ACAN expression in chondrocytes treated with incremental doses of vitamin D or K (n = 3). B. RT-qPCR analysis of MMP13, ADAMTS5, COL2A1, and ACAN expression in chondrocytes treated with vitamins D and K (n = 3). C-D. WB analysis of MMP13, ADAMTS5, COL2A1, and ACAN expression in chondrocytes treated with vitamins D and K. ns. None significant. ∗, *P* value < 0.05. ∗∗, *P* value < 0.01. ∗∗∗, *P* value < 0.001. ∗∗∗∗, *P* value < 0.0001.

RT-qPCR results revealed that only vitamin D exhibited an inhibitory effect on the upregulation of MMP13 induced by IL-1β in chondrocytes. In contrast, neither vitamin D nor K could significantly inhibit the activation of ADAMTS5 or restore the loss of COL2A1 or ACAN (Fig. 2. A). Subsequently, the effect of combined treatment of vitamins D and K on chondrocyte degradation was investigated. Vitamin D mitigated the elevated mRNA expression of MMP13 induced by IL-1β. However, it did not inhibit ADAMTS5, or recover COL2A1 or ACAN expression. On the other hand, vitamin K exhibited no significant influence on these genes, nor did it significantly enhance the inhibitory effect of vitamin D on MMP13 (Fig. 2. B). WB analysis showed similar results with RT-qPCR (Fig. 2 C-D). These findings indicated that vitamin D and K, whether administered individually or in combination, only have a limited effect on the anabolic or catabolic metabolism of OA chondrocytes. Thus, vitamins D and K might protect OA cartilage degradation via other mechanisms around the joint.

### 3.3 Vitamins D and K additively reduced osteoclast number

**Fig. 3:**
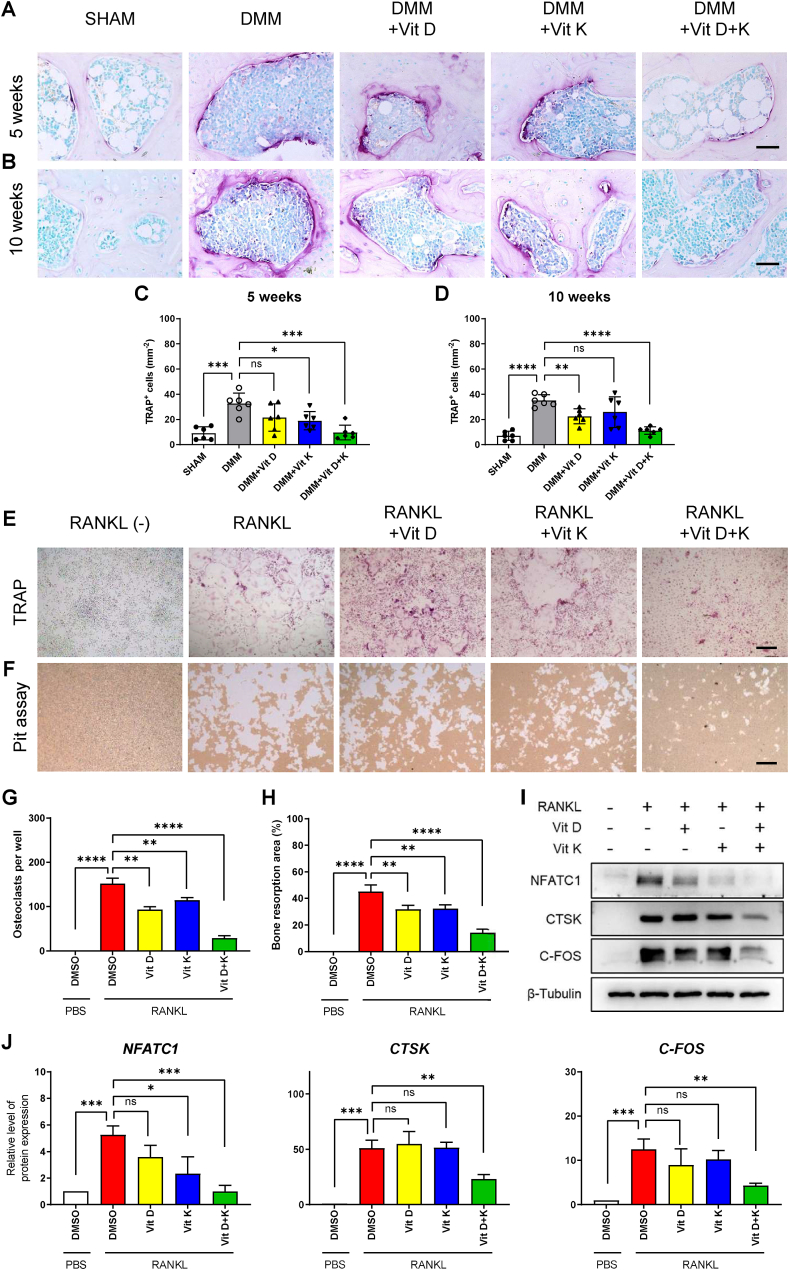
Additive inhibition on osteoclast number and osteolytic function by vitamins D and K. A-B. TRAP staining of subchondral bone 5 and 10 weeks after DMM. Scale bars, 50 μm. C-D. Quantitative analysis of osteoclast number at indicated times points (n = 6 mice per group). E. TRAP staining of RANKL-induced osteoclasts treated with vitamin D and K. Scale bar, 50 μm F. Pit assay showing resorption pit by RANKL-induced osteoclasts treated with vitamin D and K. Scale bar, 50 μm G. Quantitative analysis of osteoclast number assessed in panel E. (n = 3). H. Proportion of bone resorption area assessed in panel F (n = 3). I-J. WB analysis of NFATC1, CTSK and C-FOS expression in osteoclasts treated with vitamin D and K. ns. None significant. ∗, *P* value < 0.05. ∗∗, *P* value < 0.01. ∗∗∗, *P* value < 0.001. ∗∗∗∗, *P* value < 0.0001.

Osteoclast overactivation is demonstrated to initiate subchondral bone remodeling during OA [6,37]. Here, we aim to explore the specific role of vitamins D and K on osteoclastogenesis. TRAP staining was employed to detect osteoclast formation in the subchondral bone 5 and 10 weeks after DMM. A significant increase in osteoclast number was observed in the DMM group compared to the SHAM group, indicating that altered knee loading promoted aberrant osteoclastogenesis in the subchondral bone, in line with previous studies [38]. While vitamin D or K exhibited limited inhibition on osteoclast number in the subchondral bone, combined administration of vitamins D and K mutually enhanced the effect of each other, leading to the lowest osteoclast number in the DMM + Vit D + K group (Fig. 3 A-D). The maximum and optimal doses of vitamins D and K were determined using CCK-8 assay and TRAP staining (Figure S1 B, C). Subsequently, in vitro, we observed an enhanced suppression of osteoclast formation with the combined treatment of vitamins D and K (Fig. 3 E, G). Meanwhile, the osteolytic function of induced osteoclasts was assessed by pit assay, where combined treatment of vitamins D and K significantly reduced the osteolysis area (Fig. 3 F, H). In addition, WB analysis revealed that the combined treatment of vitamins D and K further inhibited the expression of osteoclast-marker genes, NFATC1, CTSK, and C-FOS (Fig. 3 I-J). Meanwhile, the effect of vitamin D and K on osteoblast differentiation was also investigated. After 3 or 7 days of osteogenic induction, vitamin D or K alone exerted mixed effects on the expression of these osteogenic regulators in MSCs, and the combination of vitamins D and K was inefficiently or moderately affecting the expression of these targets (Fig. S2). In all, these results strongly suggest that vitamin D and K are playing an additive inhibitory role on osteoclasts rather than osteoblasts.

### 3.4 Vitamins D and K attenuated cartilage degradation in the OPOA model

**Fig. 4:**
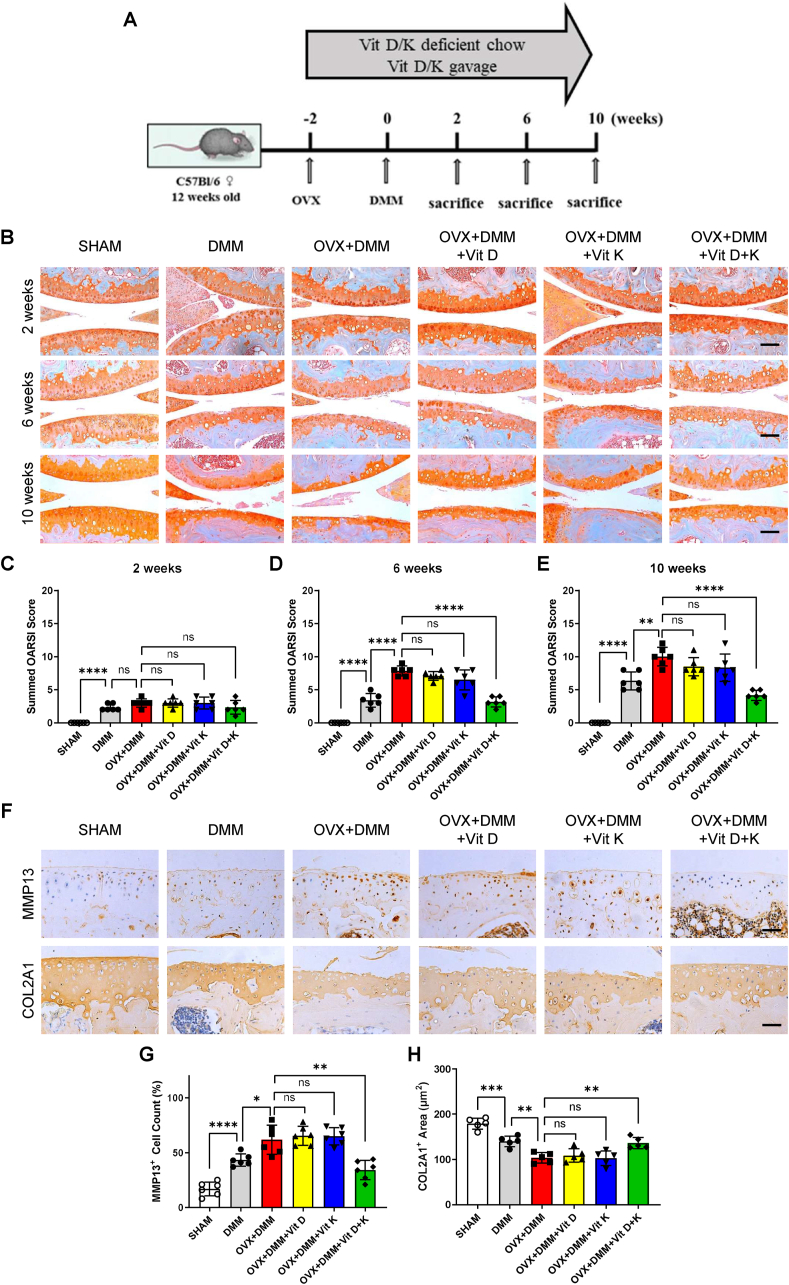
Effect of vitamins D and K on cartilage degradation in OPOA mouse models. A. Experimental scheme of vitamins D and K administration in OPOA mice. B. Safranine O/Fast green staining of articular cartilage 2, 6, and 10 weeks after OVX + DMM. Scale bar, 100 μm. C-E. Summed OARSI scoring of articular cartilage 2, 6, and 10 weeks after DMM (n = 6 mice per group). F. IHC analyses of MMP13 and COL2A1 expression in the articular cartilage 6 weeks after OVX + DMM. G. The percentage of MMP13-positive cells in the articular cartilage assessed in panel F (n = 6 mice per group). H. Area of COL2A1-positive extracellular matrix of the articular cartilage assessed in panel F (n = 6 mice per group). ns. None significant. ∗, *P* value < 0.05. ∗∗, *P* value < 0.01. ∗∗∗, *P* value < 0.001. ∗∗∗∗, *P* value < 0.0001.

Osteoporosis (OP) contributes partly to OA progression in human and animal models by promoting osteoclastogenesis and thus results in abnormal subchondral bone remodeling [[17], [39]]. Based on their joint inhibition of osteoclastogenesis, we investigated the effect of vitamins D and K on OPOA models, where osteoclasts were overactivated in the subchondral bone. To do so, we performed OVX 2 weeks before DMM. Meanwhile, vitamins D and K were orally given from 2 weeks prior to DMM to 10 weeks after DMM (Fig. 4. A). At two weeks post-DMM, although mild cartilage degradation was induced in DMM and OVX + DMM groups, no significant differences in cartilage degradation were observed between model and treatment groups (Fig. 4. C). Interestingly, at 6 and 10 weeks post-DMM, mice in the OVX + DMM group experienced accelerated cartilage degradation compared to DMM group, while combined treatment of vitamins D and K attenuated cartilage degradation more effectively than vitamin D or K alone (Fig. 4 D, E). Furthermore, chondrocyte catabolism and ECM composition were detected by IHC staining. Compared to DMM group, OVX + DMM group exerted significantly increased ratio of MMP13 positive cells and decreased area of COL2A1 positive ECM. Consistently, combined supplementation of vitamins D and K significantly alleviated cartilage degradation, while vitamin D or K alone could not (Fig. 4 F-H). These results demonstrated that cartilage degradation in OPOA mice was significantly attenuated by the combined treatment of vitamins D and K.

### 3.5 Vitamins D and K normalized subchondral bone remodeling in OPOA mice

**Fig. 5:**
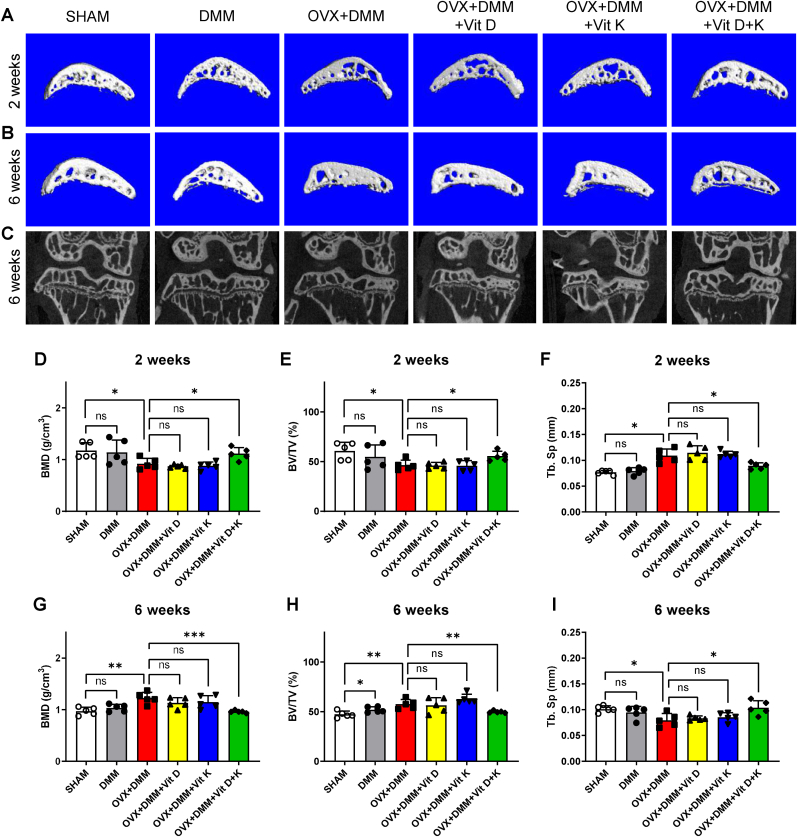
Effect of vitamins D and K on subchondral bone microstructural changes in OPOA mouse models. A-B. Representative microstructures of medial tibial subchondral bone 2 and 6 weeks after OVX + DMM. C. Coronal view of knee joint structures 6 weeks after OVX + DMM. D-F. BMD, BV/TV, and Tb. Sp analysis of subchondral bone 2 weeks after OVX + DMM (n = 5 mice per group). G-I. BMD, BV/TV, and Tb. Sp analysis of subchondral bone 6 weeks after OVX + DMM (n = 5 mice per group). ns. None significant. ∗, *P* value < 0.05. ∗∗, *P* value < 0.01. ∗∗∗, *P* value < 0.001.

Subsequently, the impact of vitamins D and K on subchondral bone remodeling was assessed using μ-CT analysis. Our findings revealed that the subchondral bone of OPOA mice experienced dynamic changes. At 2 weeks after DMM, which can be considered an early stage of OPOA, subchondral bone exhibited osteoporotic phenotypes characterized by larger separation zone between trabecular bones in the OVX + DMM group, compared to those in the SHAM and DMM groups. However, mice treated with vitamins D and K exhibited comparable morphology with those in the SHAM group, while those treated with vitamin D or K alone did not (Fig. 5 A-C). Structural parameter analysis showed consistent trends with morphological features. Decreased bone mineral density (BMD) and bone volume fraction (BV/TV) and increased trabecular separation (Tb. Sp) were observed in the OVX + DMM group, compared to the SHAM and DMM groups. Meanwhile, combined treatment of vitamins D and K resulted in parameters comparable to those in the SHAM and DMM groups, while single use of either vitamin alone did not (Fig. 5 D-F). Intriguingly, at six weeks after DMM, subchondral bone in the OVX + DMM group transitioned from osteoporotic phenotypes towards sclerotic phenotypes, characterized by solid, plate-like morphology (Fig. 5 B, C). Consistently, increased BMD, BV/TV and decreased Tb. Sp were observed in the OVX + DMM group, compared to the SHAM and DMM groups, while combined treatment of vitamins D and K managed to maintain a comparable level of these parameters with the SHAM group (Fig. 5 G-I). These results indicated that subchondral bone remodeling during OA was accelerated by OVX, and combined treatment of vitamins D and K effectively normalized these phenotype changes.

### 3.6 Vitamins D and K jointly regulated subchondral bone metabolism

**Fig. 6:**
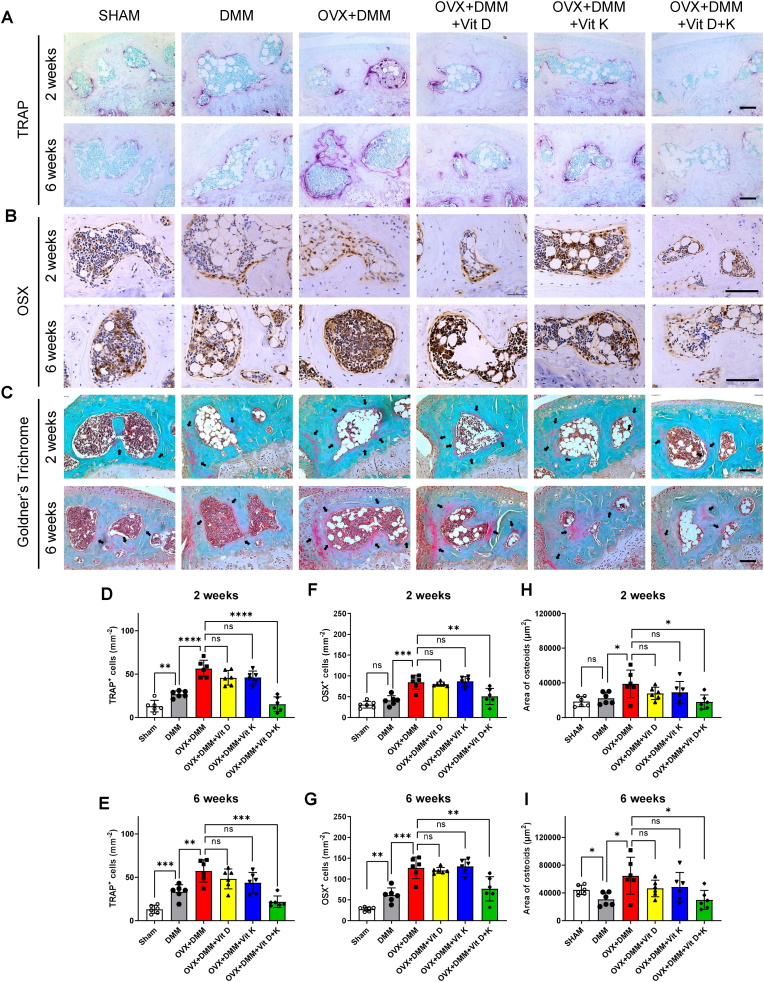
Effect of vitamins D and K on subchondral bone metabolism in OPOA mouse models. A. TRAP staining of osteoclasts in subchondral bone 2 and 6 weeks after DMM. B. IHC staining of OSX in subchondral bone 2 and 6 weeks after DMM. C. Goldner's trichrome staining shows newly formed bone tissue in subchondral bone 2 and 6 weeks after DMM. D-E. Quantification analysis of osteoclast number assessed in panel A (n = 6 mice per group). F-G. Quantification analysis of OSX positive cells assessed in panel B (n = 6 mice per group). H-I. Quantification analysis of newly formed bone area assessed in panel C (n = 6 mice per group). ns. None significant. ∗, *P* value < 0.05. ∗∗, *P* value < 0.01. ∗∗∗, *P* value < 0.001. ∗∗∗∗, *P* value < 0.0001.

Next, we examined the specific role of vitamins D and K in subchondral bone metabolism in OPOA mice. TRAP staining showed that subchondral bone osteoclastogenesis was significantly promoted in the OVX + DMM group, with the highest osteoclast number at 2 and 6 weeks after DMM, while vitamins D and K, rather than either one alone, effectively suppressed these changes (Fig. 6 A, D, E). Meanwhile, IHC staining of osterix (OSX), a landmark of osteoblast, revealed that osteoblasts were significantly induced and increased with time in the OVX + DMM group, compared to SHAM and DMM groups. However, combined treatment of vitamins D and K suppressed these changes, resulting in an osteoblast number comparable to the SHAM group (Fig. 6 B, F, G). Furthermore, by Goldner's trichrome staining, we captured the bone formation process, indicated by the pink staining on subchondral bones, during OPOA progression (Fig. 6. C). Specifically, at two weeks after DMM, a low level of bone formation was observed in the OVX + DMM group compared to the SHAM group, and combined treatment of vitamins D and K effectively suppressed this change, where vitamin D or K alone could not (Fig. 6 H). Intriguingly, at six weeks after DMM, compared to the SHAM and DMM groups, a significant increase in bone formation area was observed in the OVX + DMM group, and combined treatment of vitamins D and K significantly inhibited these changes, while single treatment of vitamin D or K did not (Fig. 6 I). These results demonstrated that subchondral bone turnover and formation were effectively stabilized by the combined treatment of vitamins D and K via their joint inhibition of osteoclastogenesis.

### 3.7 Vitamins D and K relieved pain in OPOA mice

**Fig. 7:**
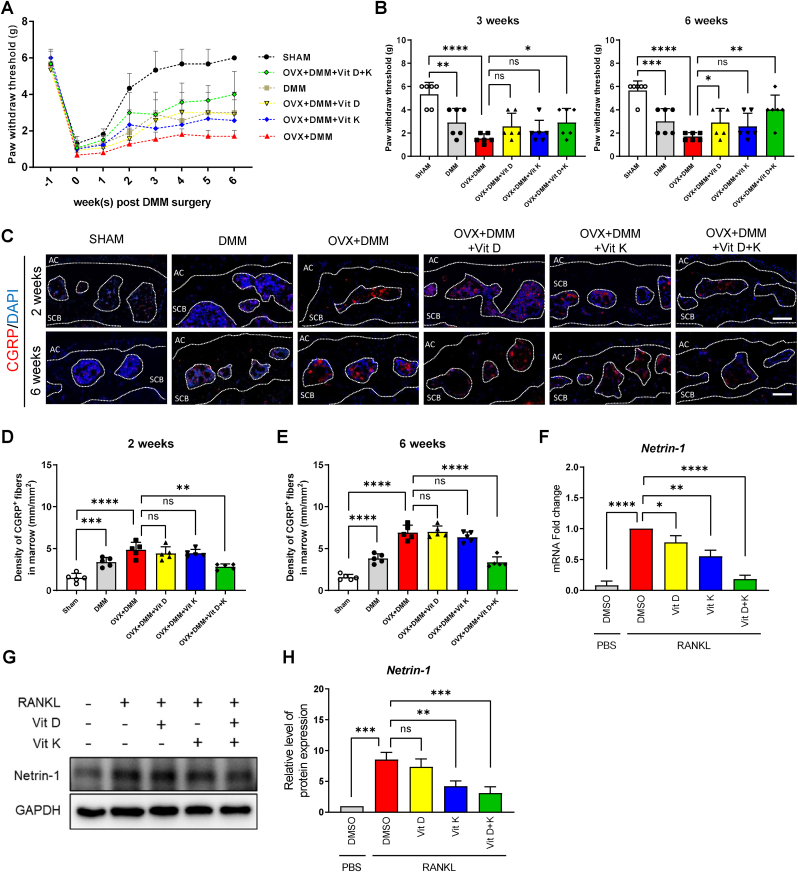
Effect of vitamins D and K on pain and sensory innervation in OPOA mouse models. A. Paw withdraw threshold assessment by von-Frey test (n = 6 mice per group). B. Cross-sectional analysis of PWT at 3 and 6 weeks after DMM (n = 6 mice per group). C. Immunofluorescence staining of CGRP in subchondral bone at 2 and 6 weeks after DMM. Scale bars, 50 μm. D-E. Quantitative analysis of the density of CGRP-positive fibers in the subchondral bone at 2 and 6 weeks after DMM (n = 5 mice per group). F. RT-qPCR analysis of Netrin1 in osteoclasts treated with vitamins D and K. G-H. WB analysis of Netrin1 in osteoclasts treated with vitamins D and K. ns. None significant. ∗, *P* value < 0.05. ∗∗, *P* value < 0.01. ∗∗∗, *P* value < 0.001. ∗∗∗∗, *P* value < 0.0001.

The process of subchondral bone remodeling has been proven to be accompanied by sensory innervation, which can aggravate pain sensitization in lower limbs [40,41]. Here, we assessed pain levels in OPOA mice using the Von Frey test as previously described [42]. We found no significant differences in paw withdraw threshold (PWT) between groups at baseline (1 Week prior to DMM). On the second day after DMM (Week 0), the PWT of all groups decreased dramatically, which was attributable to acute pain caused by surgical trauma. During subsequent weekly follow-up, the PWT of each group recovered at different rates (Fig. 7. A). At three weeks after DMM, the PWT of the OVX + DMM group was significantly lower than those of SHAM and DMM groups, while combined treatment of vitamins D and K effectively promoted the recovery of PWT, and this difference was more evident at six weeks after DMM. However, treatment of vitamin D or K alone only exerted limited therapeutic effect, with no significant improvement in pain level (Fig. 7. B). By immunofluorescence staining, calcitonin gene-related peptide (CGRP), a marker of nociceptive nerve fibers, was found to be significantly upregulated in the subchondral bone of OVX + DMM group at two weeks after DMM, with a further increase at six weeks after DMM. Intriguingly, combined treatment of vitamins D and K reduced CGRP^+^ sensory innervation, while vitamin D or K alone could not (Fig. 7 C-E). In addition, in previous studies, an increased level of Netrin-1 was observed in overactivated osteoclasts, which induces sensory innervation into the subchondral bones and results in OA-related pain. Furthermore, Netrin-1 knock-out mice experienced attenuated OA pain [40,43]. In the present study, by RT-qPCR and WB, we also found that RANKL-induced osteoclasts expressed a higher level of Netrin-1, which was significantly inhibited by the combined treatment of vitamins D and K in vitro (Fig. 7 F-H). The above data implied that vitamins D and K effectively alleviate pain and nociceptive innervation partly via joint inhibition of Netrin-1 derived from overactivated osteoclasts.

### 3.8 OSCAR regulates osteoclastogenesis and mediates the joint inhibition of vitamins D and K on osteoclasts

**Fig. 8:**
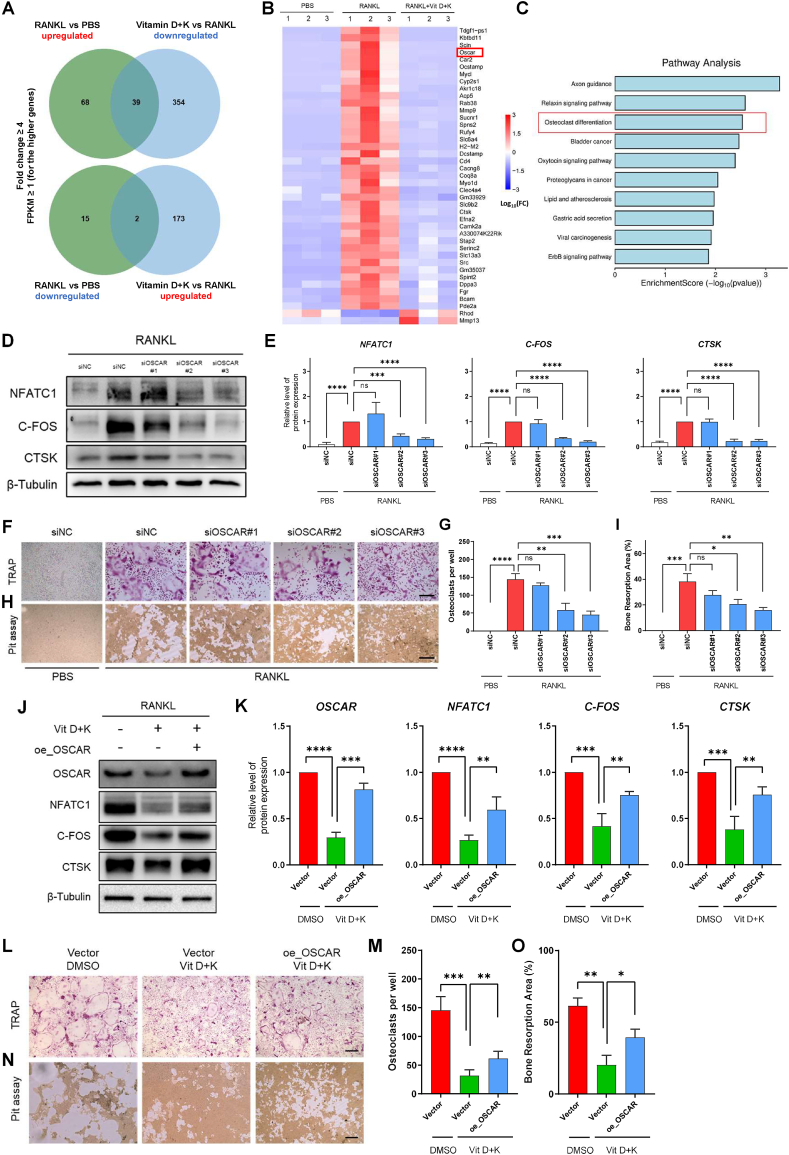
Vitamins D and K inhibited osteoclastogenesis via joint inhibition of OSCAR. A. Venn blot showing the screening strategy of differentially expressed genes. B. Heat map analysis of differentially expressed genes in panel A. C. KEGG enrichment analysis of differentially expressed genes in panel A. D-E. WB analysis of OSCAR (n = 3). E. WB analysis of osteoclast marker proteins NFATC1, CTSK, and C-FOS after OSCAR knockdown. F. TRAP staining of osteoclasts after OSCAR knockdown. Scale bar, 50 μm G. Quantitative analysis of osteoclast number assessed in panel F (n = 3). H. Pit assay showing bone resorption pits by osteoclasts after OSCAR knockdown. Scale bar, 50 μm. I. Proportion of osteolysis area assessed in panel H (n = 3). J-K. WB analysis of OSCAR, NFATC1, CTSK, and C-FOS after OSCAR overexpression and treatment with vitamins D and K. L. TRAP staining of osteoclasts after OSCAR overexpression and treatment with vitamins D and K. Scale bar, 50 μm M. Quantitative analysis of osteoclasts number assessed in panel L (n = 3). N. Pit assay showing bone resorption pits by osteoclasts after OSCAR overexpression and treatment with vitamins D and K. Scale bar, 50 μm. O. Proportion of osteolysis area assessed in panel N (n = 3). ns. None significant. ∗, *P* value < 0.05. ∗∗, *P* value < 0.01. ∗∗∗, *P* value < 0.001. ∗∗∗∗, *P* value < 0.0001.

We performed RNA sequencing on preosteoclasts from three experimental groups: PBS group, RANKL group, and RANKL + Vit D + K group. Through rigorous screening, we identified 39 potential pro-osteoclastic genes that were downregulated by vitamin D and K intervention (exhibiting significant upregulation in the RANKL group compared to both PBS and RANKL + Vit D + K groups with fold change ≥4, and FPKM ≥1). Concurrently, 2 putative anti-osteoclastic genes showed marked upregulation upon vitamin D and K treatment (demonstrating significant downregulation in the RANKL group versus both PBS and RANKL + Vit D + K groups with fold change ≥4, and FPKM ≥1 in both PBS and RANKL + Vit D + K groups) (Fig. 8 A, B). KEGG pathway enrichment analysis of these differentially expressed genes revealed significant clustering of OSCAR, Acp5 (TRAP), and CTSK within the “osteoclast differentiation” pathway (Fig. 8. C). As OSCAR is located upstream of Acp5 and CTSK, two well established osteoclast regulators, in the "osteoclast differentiation" pathway, we further investigated whether OSCAR could mediate the inhibitory effect of vitamin D and K on osteoclastogenesis. By validation with RT-qPCR and WB analysis, we find the expression patterns of OSCAR consistent with the RNA sequencing data (Figure S3 A-C). To verify the role of OSCAR on osteoclastogenesis, OSCAR-specific small interfering siRNAs (siOSCAR) were applied to knock down the mRNA expression of OSCAR, and their interference efficiency was verified by RT-qPCR assay (Figure S3 D). Consequently, protein expression levels of osteoclast regulative genes NFATC1, C-FOS, and CTSK were significantly suppressed by OSCAR knockdown (Fig. 8 D-E). Moreover, the knockdown of OSCAR significantly reduced osteoclast number and their bone resorptive activity (Fig. 8 F-I). These results indicate that OSCAR is crucial for osteoclastogenesis. Next, we aimed to determine whether vitamins D and K could inhibit osteoclastogenesis by targeting OSCAR. By over-expressing OSCAR in preosteoclasts, we found that the inhibition of vitamins D and K, either alone or in combination on osteoclast regulative proteins NFATC1, C-FOS, and CTSK was partially antagonized (Fig. 8 J, K; Figure S4 A, B). Also, by TRAP staining and pit assay, we observed consistent trends that OSCAR over-expression partially restored osteoclast number and osteolysis area, which were reduced by vitamins D and K (Fig. 8 L-O). Together, these results suggested that vitamins D and K may inhibit osteoclastogenesis and their osteolytic function by, at least in part, jointly regulating OSCAR in osteoclast precursors.

## 4 Discussion

To our knowledge, we revealed for the first time the role of combined treatment of vitamins D and K in OA and OPOA progression. Vitamins D and K both play essential roles in the incidence and progression of OA. However, supplementation of either one alone only achieved limited benefit for OA patients, leading to ongoing controversy regarding their clinical significance in OA treatment. Previous studies have predominantly focused on their separated efficacy while neglecting their combined therapeutic effects, particularly in chronic musculoskeletal disorders such as OA and its subtype, OPOA. In this study, we demonstrated that vitamins D and K effectively retarded OA and OPOA progression. Specifically, vitamins D and K jointly preserved the microstructure of subchondral bone and attenuated joint pain, presumably by suppressing osteoclast-related sensory innervation in the subchondral bone. Simultaneously, the deterioration of articular cartilage was significantly alleviated by vitamins D and K both in OA and OPOA models with limited direct effect on OA chondrocytes. Mechanistically, vitamins D and K effectively inhibited the excessive osteoclastogenesis probably through joint inhibition of OSCAR in preosteoclasts.

The immediate effects of vitamin D or K on OA chondrocytes have been studied, yet many remain unrevealed. Vitamin D is reported to exert mixed effects on chondrocyte degeneration. In 2001, Tetlow et al. found that, in vitro, 1,25(OH)_2_D_3_ upregulated MMP3, an ECM degradative enzyme, in inflamed chondrocytes induced by TNF-α or phorbol myristate acetate (PMA); meanwhile, 1,25(OH)_2_D_3_ inhibited the expression of another ECM degradative enzyme, MMP9 and an inflammatory cytokine, PGE2 induced by PMA [44]. Recently, Chen et al. established a genetically modified 1,25(OH)_2_D_3_ deficient mice by knocking out 1α(OH)ase in the kidney and found that these mice experienced aggravated aging-related spontaneous OA as a result of down-regulated Sirt1 signaling in chondrocytes [45]. As for vitamin K, studies mainly focused on the anti-mineralization of certain vitamin K-dependent proteins (VKDPs), whose function depends on the carboxylation of vitamin K-related enzymes [46]. Wallin et al. found that chondrocytes from OA cartilage produced much less carboxylated MGP, a VKDP associated with anti-mineralization, than those from healthy cartilage, which is in line with the fact that OA chondrocytes exert weaken vitamin K-dependent gamma-carboxylase enzyme activity [47]. Additionally, Cavaco et al. found that exogenous Gla-rich protein (GRP), another VKDP, could protect from cartilage mineralization in OA [48]. In our study, only combined administration of vitamins D and K effectively prevented cartilage degradation in OA and OPOA models, whereas either vitamin alone failed to provide sufficient protection. Furthermore, except for a moderate suppression of MMP13 by calcitriol, neither calcitriol nor MK4 was able to inhibit the increased expression of ADAMTS5 or restore the levels of COL2A1 and ACAN. Additionally, MK4 could not enhance the efficacy of calcitriol. These findings suggest that vitamins D and K provide limited protection for OA chondrocytes, indicating that their therapeutic effects may operate through alternative mechanisms.

Aberrantly activated osteoclastogenesis leads to pathological changes of subchondral bone and progressively induces sensory innervation and angiogenesis within the subchondral bone marrow, resulting in pain sensitization and altered microenvironment [8]. In the advanced stages of OA, TGF-β1 released from the bone matrix induces an overabundance of osteoblasts as a consequence of osteoclast-mediated bone resorption during the early phases of OA. This remodeling process transitions from osteoporotic changes to increased bone formation and sclerosis, ultimately impairing the biomechanical properties of the subchondral bone and exacerbating damage to the overlying articular cartilage [3]. Therefore, it is hypothesized that pharmacological agents targeting osteoclastogenesis, particularly in the early phase of OA, may represent a promising therapeutic strategy [13].

As two critical bone-modulating agents, the precise effects of vitamins D and K on osteoclastogenesis remain to be fully elucidated. Vitamin D has been reported to exert a biphasic regulatory effect on osteoclastogenesis. Li et al. reported that 1,25(OH)_2_D_3_ can either inhibit or promote osteoclast formation, depending on whether 1,25(OH)_2_D_3_ was administered prior to or following RANKL stimulation. Mechanistically, the inhibition of osteoclastogenesis occurs through modulation of the BMP-Smad1 pathway, whereas enhancement is mediated by activation of the IκB-NF-κB pathway [49]. Similarly, Ji et al. found that in the absence of RANKL, 1,25(OH)_2_D_3_ directly suppressed the autophagy level of preosteoclasts, resulting in suppressed cell proliferation. Meanwhile, in the presence of RANKL, 1,25(OH)_2_D_3_ indirectly upregulated the autophagy response of preosteoclasts, thereby enhancing osteoclastogenesis [50]. Additionally, vitamin K is reported to suppress bone resorption in the OVX-induced osteoporosis mouse model [51] and inhibit osteoclastogenesis via inhibition of NF-κB signaling [52]. In this study, we observed a significantly enhanced inhibition of osteoclastogenesis by the combined administration of calcitriol and MK4, both in vivo and in vitro. Specifically, compared to monotherapy, the combination treatment with vitamins D and K resulted in greater suppression of RANKL-induced expression of CTSK, C-FOS, and NFATC1 in preosteoclasts. Furthermore, this combination therapy effectively normalized the exacerbated subchondral bone remodeling observed in OPOA models, whereas monotherapy did not yield similar results.

Abnormal sensory innervation resulting from osteoclast activation in the subchondral bone contributes to OA pain [43]. Specifically, osteoclasts secrete Netrin-1, promoting the infiltration of CGRP-positive sensory nerves into the vertebral pulp and subchondral bone marrow, which results in pain sensitization [10,43]. In this study, we observed that vitamins D and K significantly inhibited the mRNA expression of Netrin-1 in osteoclasts. In vivo, compared to the SHAM and DMM groups, CGRP-positive fibers in the subchondral bone were significantly elevated in the OVX + DMM group, which was markedly inhibited by the combined treatment of vitamins D and K; however, monotherapy with either vitamin alone proved ineffective. Consistently, the combined administration of vitamins D and K led to a significant recovery of the paw withdrawal threshold of the modeled mice, indicating its potential for pain relief.

For cellular mechanisms, we found that vitamins D and K significantly suppressed osteoclastogenesis by joint inhibition of OSCAR in preosteoclasts. During RANKL-induced osteoclastogenesis, mononucleated preosteoclasts derived from monocytes or macrophages fuse to form multinucleated osteoclasts, acquiring osteolytic function. RNA sequencing analysis and subsequent validations in preosteoclasts revealed that OSCAR was upregulated by RANKL and was jointly suppressed by vitamins D and K. OSCAR is a collagen receptor that co-stimulates osteoclastogenesis with DAP12 [47]. In this study, we observed that inhibition of OSCAR in preosteoclasts significantly reduced osteoclast number and their resorptive activity, while OSCAR effectively mitigated the inhibitory effects of the combined vitamin D and K treatment.

With similar mechanism to vitamins D and K combination, bisphosphonates are also potential candidates for OA treatment. However, bisphosphonates are associated with side effect, limiting their use in certain populations, particularly those with renal impairment or gastrointestinal disorders [53]. In contrast, vitamin D and K both have a high safety profile and is suitable for a broader population. Beyond bone health, vitamin D and K provide potential multisystem benefits, such as improving cardiovascular health and reducing inflammation [54]. Economically, vitamin D and K are generally more affordable, making it a cost-effective option with positive socioeconomic implications, especially for long-term use in large populations. In all, combination of vitamin D and K could serve as a promising alternative in managing bone-related disorders, such as OA and OPOA.

In conclusion, vitamins D and K exerted joint effects on disease progression of OA and its subtype, OPOA, through inhibiting osteoclastogenesis by regulating OSCAR in preosteoclasts. Based on current data, vitamins D and K, as a whole, represent a promising pharmacological candidate for preventing and treating OA, especially OPOA. Further investigation of vitamins D and K, such as RCTs, would provide more supportive evidence on their therapeutic significance for OA.

There are several limitations of this study. Firstly, the mixed effects of vitamin D on chondrocyte degradation and the anti-mineralization role of vitamin K in cartilage homeostasis suggest a complex interaction that was not fully elucidated in this study. Secondly, although the study provides valuable insights through its reliance on animal models, it may not fully capture the complexity of human OA and OPOA. The application of these findings to human subjects necessitates further investigation, including RCTs, to validate the therapeutic significance of vitamins D and K in OA treatment. Lastly, although we have identified OSCAR as a pivotal regulator in osteoclastogenesis that is jointly inhibited by vitamins D and K, the broader molecular mechanisms governing this regulation and their implications for OA pain and progression warrant further elucidation.

## 5 Conclusion

This study provides novel insights into the joint effect of vitamins D and K on the progression of OA and its subtype, OPOA. In addition, Vitamins D and K collaborate to inhibit osteoclastogenesis by regulating OSCAR in preosteoclasts, so facilitating the restoration of subchondral bone remodeling and relieving pain throughout the disease progression. Hence, the combined administration of vitamin D and K is expected to be a potential therapy for OA, specifically for its subtype, OPOA, where subchondral bone remodeling is aggravated. Future clinical studies comparing the vitamin D/K combination with established antiresorptive therapies, such as bisphosphonates and denosumab, would be valuable for assessing this relative efficacy of the natural and potentially safer vitamin formulation for OA treatment.

## Ethics approval and consent to participate

All operational procedures conformed to the requirements and guidelines of the ethics committee of Zhujiang Hospital (LEAC-2020-021). The articular cartilage was obtained from patients with OA who had undergone knee replacement in the Department of Orthopedics, Zhujiang Hospital, Southern Medical University. This study was approved by the Ethics Committee of Zhujiang Hospital, Southern Medical University (2019KY02203).

## Consent for publication

Not applicable.

## Availability of data and material

The RNA-Seq data of preosteoclasts stimulated with RANKL and vitamin D and K are available in the GEO database (accession number GSE272401).

## Fundings

The study was funded by the 10.13039/501100001809National Natural Science Foundation of China (82373653, 82472478), the 10.13039/501100021171Basic and Applied Basic Research Foundation of Guangdong Province (2023A1515011518).

## Declaration of competing interest

The authors declare no competing interests.
