# Supplementary figures and images for: Vitamins D and K jointly protect against osteoarthritis via regulating OSCAR during osteoclastogenesis
Source: J Orthop Translat. 2025 May 12;52:387–403. doi: 10.1016/j.jot.2025.03.018 (PMC12137181; doi:10.1016/j.jot.2025.03.018)

**A**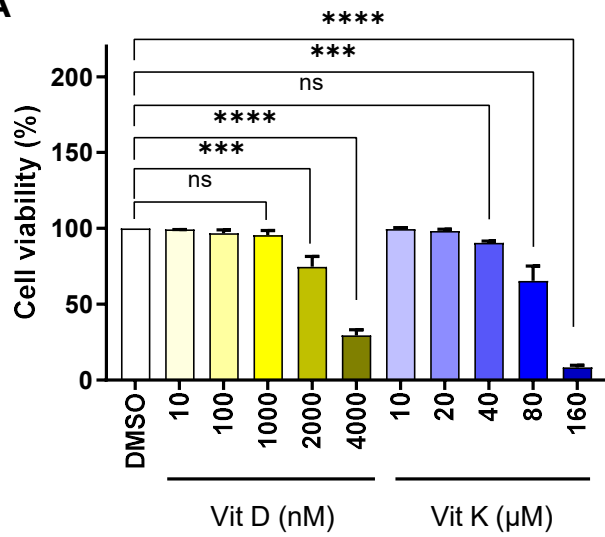**B**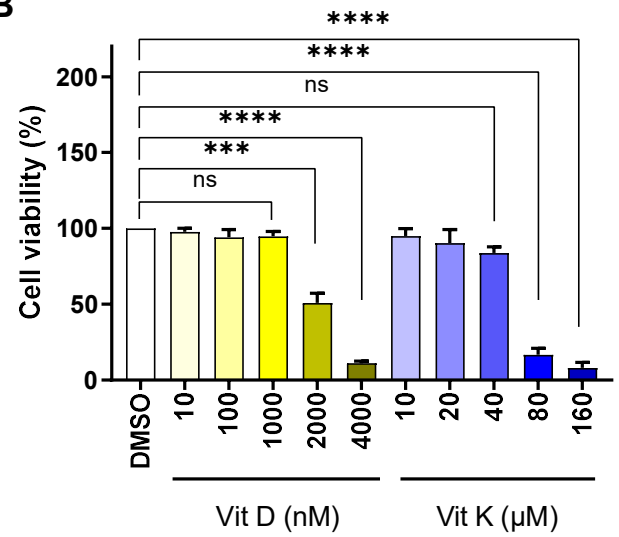**C**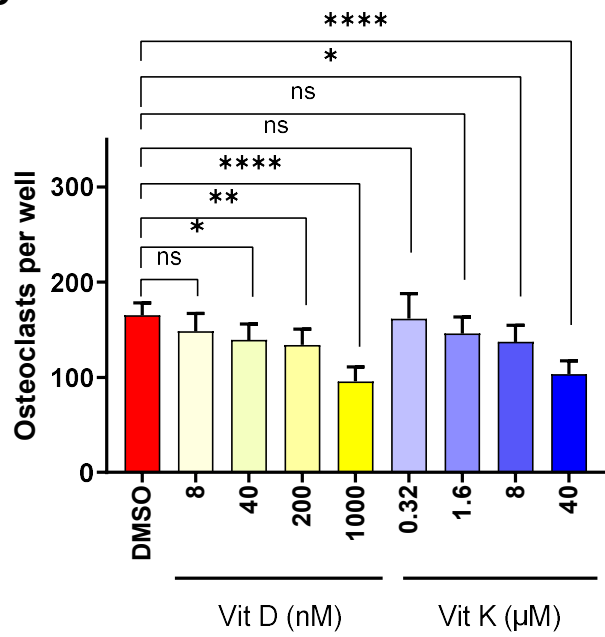

Supplement: Multimedia component 1 [file mmc1.pdf]

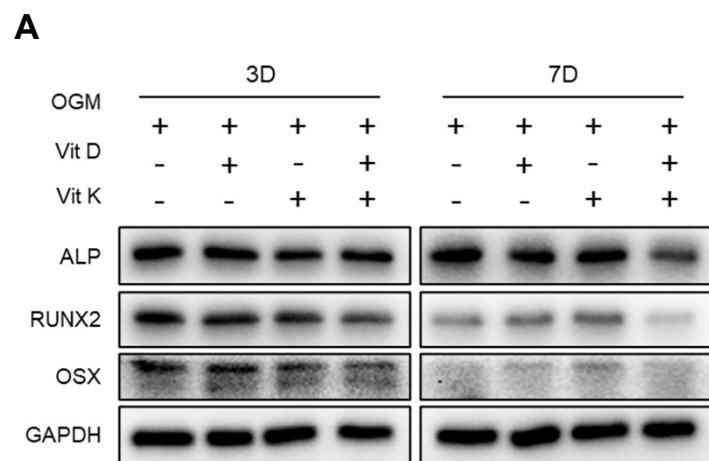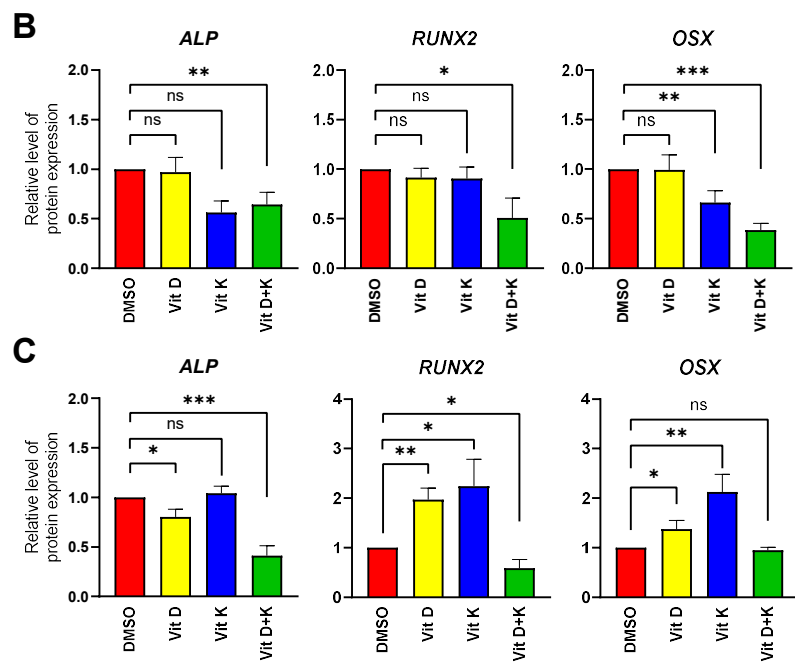

Supplement: Multimedia component 2 [file mmc2.pdf]

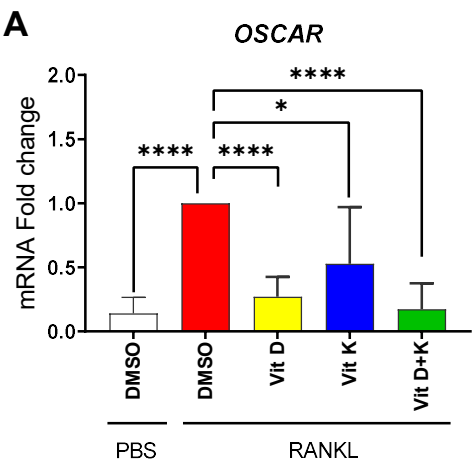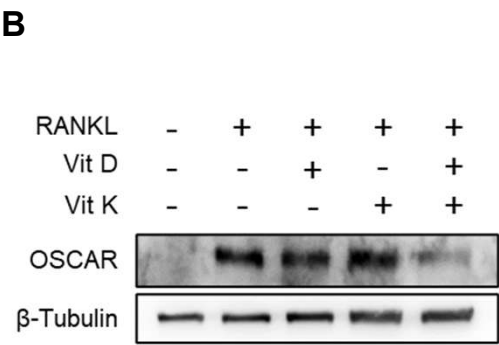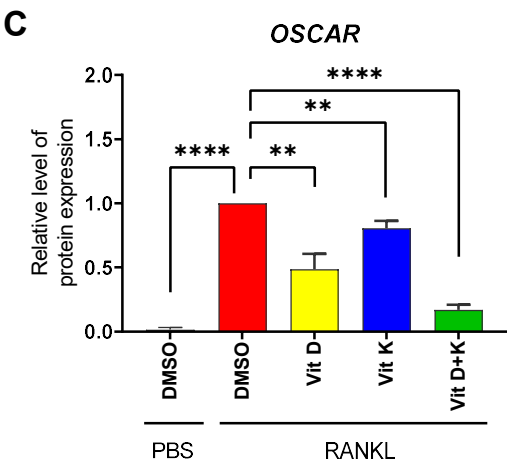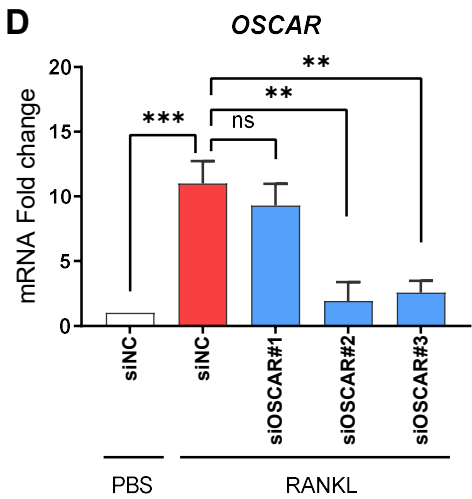

Supplement: Multimedia component 3 [file mmc3.pdf]

**A**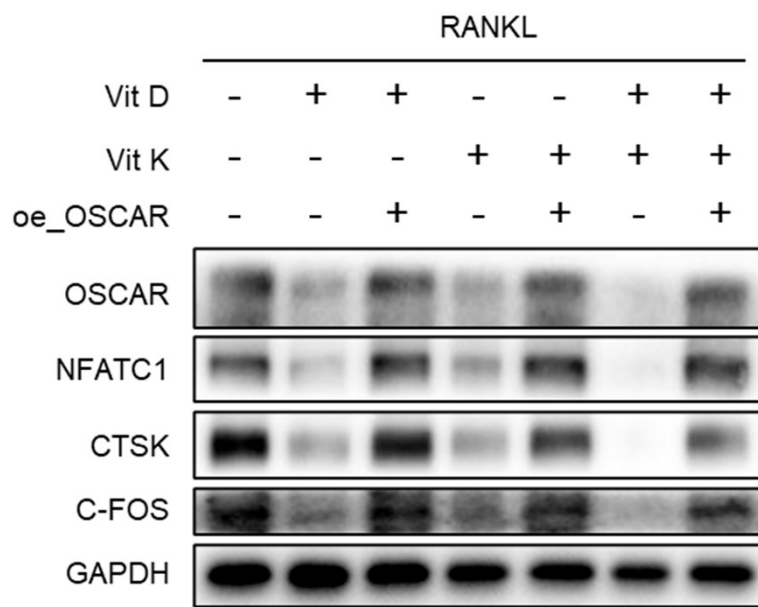**B**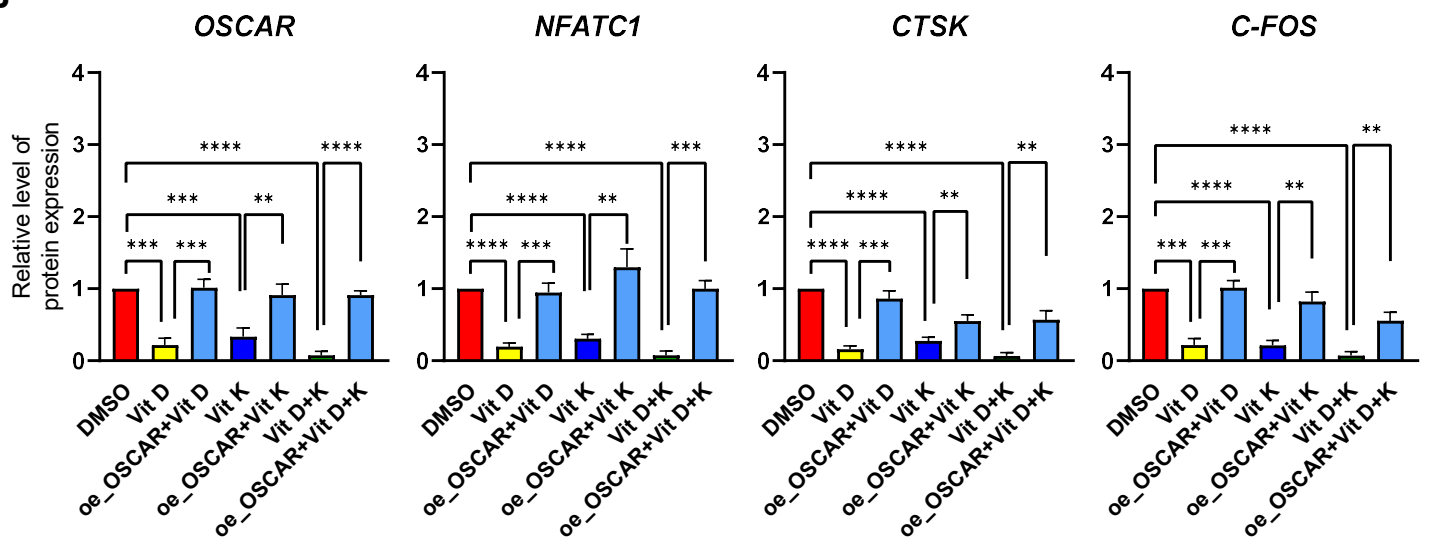

Supplement: Multimedia component 4 [file mmc4.pdf]
