# Supplementary material for: Vitamins D and K jointly protect against osteoarthritis via regulating OSCAR during osteoclastogenesis
Source: J Orthop Translat. 2025 May 12;52:387–403. doi: 10.1016/j.jot.2025.03.018 (PMC12137181; doi:10.1016/j.jot.2025.03.018)
Supplement: Multimedia component 5 [file mmc5.docx]

**Table S1. Primer pairs used in this study**

A. Human primers used for RT-qPCR

| Gene | Forward primer (5’-3’) | Reverse primer (5’-3’) |
| --- | --- | --- |
| GAPDH | CTGGGCTACTACTGAGCACC | AAGTGGTCGTTGAGGGCAATG |
| MMP13 | GACTTCCCAGGAATTGGTGA | TGACGCGAACAATACGGTTA |
| ADAMTS5 | GAACATCGACCAACTCTACTCCG | CAATGCCCACCGAACCATCT |
| COL2A1 | TGGACGCCATGAAGGTTTTCT | TGGGAGCCAGATTGTCATCTC |
| ACAN | GTGCCTATCAGGACAAGGTCT | GATGCCTTTCACCACGACTTC |

B. Mouse primers used for RT-qPCR

| Gene | Forward primer (5’-3’) | Reverse primer (5’-3’) |
| --- | --- | --- |
| GAPDH | CTGGGCTACTACTGAGCACC | AAGTGGTCGTTGAGGGCAATG |
| Oscar | TTGCAAAGCCTGTGATTGCC | GCCTCTCAGGACCTCCCTAA |
| Netrin-1 | GTCGCTCGGCAAGAAGTTTG | TGTACATTTTGCGGCACTGC |
