# Supplementary material for: Vitamins D and K jointly protect against osteoarthritis via regulating OSCAR during osteoclastogenesis
Source: J Orthop Translat. 2025 May 12;52:387–403. doi: 10.1016/j.jot.2025.03.018 (PMC12137181; doi:10.1016/j.jot.2025.03.018)
Supplement: Multimedia component 6 [file mmc6.docx]

**Table S2. siRNA sequences of OSCAR**

| siRNA | Forward sequence (5’-3’) | Reverse sequence (5’-3’) |
| --- | --- | --- |
| siOSCAR#1 | GCAUCAUAGUUACUUGUGAUUTT | AAUCACAAGUAACUAUGAUGCTT |
| siOSCAR#2 | CAGCCCAGUAAUGUUCUGGAATT | UUCCAGAACAUUACUGGGCUGTT |
| siOSCAR#3 | CUUGGACUAUACCCAGGGAAATT | UUUCCCUGGGUAUAGUCCAAGTT |
| siNC | UUCUCCGAACGUGUCACGUdTdT | ACGUGACACGUUCGGAGAAdTdT |
