# Supplementary material for: Vitamins D and K jointly protect against osteoarthritis via regulating OSCAR during osteoclastogenesis
Source: J Orthop Translat. 2025 May 12;52:387–403. doi: 10.1016/j.jot.2025.03.018 (PMC12137181; doi:10.1016/j.jot.2025.03.018)
Supplement: Multimedia component 7 [file mmc7.docx]

**Table S3. Basic clinical information of patients donating cartilage samples.**
